# Complete genome sequence of *Actinoplanes sichuanensis* strain 03-723^T^

**DOI:** 10.1128/MRA.00662-23

**Published:** 2023-11-14

**Authors:** Taketsugu Kawashima, Yasuha Watanabe, Kazuharu Arakawa

**Affiliations:** 1 University of Toronto, Faculty of Applied Science and Engineering, Toronto, Ontario, Canada; 2 Institute for Advanced Biosciences, Keio University, Tsuruoka, Yamagata, Japan; 3 Keio University, Faculty of Environment and Information Studies, Fujisawa, Kanagawa, Japan; California State University San Marcos, San Marcos, California, USA

**Keywords:** *Actinoplanes*, acarbose

## Abstract

A mesophilic bacterium *Actinoplanes sichuanensis* strain 03-723**^T^** was previously isolated from soil by Sun et al. Here, we present a complete and annotated genome sequence of this strain, which has a total size of 12.1 Mbp with a G + C content of 70.1%.

## ANNOUNCEMENT

Members of the genus *Actinoplanes,* a Gram-positive bacteria, are natural producers of acarbose, an alpha-glucosidase inhibitor widely employed in treating diabetes mellitus type II (1, 2). Given the increasing global prevalence of diabetes mellitus type II (3), there is a growing need for a better understanding of acarbose production. To address this need, we report the complete genome sequence of *Actinoplanes sichuanensis* to analyze its potential in acarbose production.

*Actinoplanes sichuanensis* strain 03-723**^T^** was obtained from the Japan Collection of Microorganisms, RIKEN BRC, and subcultured aerobically at 30°C for 3 days using a liquid medium composed of yeast extract (200 µg/100 mL), soluble starch (1 mg/100 mL), and distilled water (remainder) with an adjusted pH of 7.3 (4). Genomic DNA was extracted using the NucleoBond HMW DNA Kit (Takara Bio) and gave a TapeStation result of main peaks at >60,000 bp. Confirmation of an appropriate size for sequencing has been ensured, and no size selection has been performed. Subsequently, a long-read sequencing library was prepared according to Ligation Sequencing Kit V14 and was sequenced using PromethION Flow Cell R10.4.1 on P2 Solo device (Oxford Nanopore Technologies). The obtained data were base called using Guppy version 6.4.6 with the “super accurate” basecalling mode, and the adapters were trimmed.

Sequencing yielded a total of 2.477 Gbp long reads (N_50_, 8,594 bp) constructed from 604,542 reads produced with an estimated coverage of 100× after filtering the reads over 10 kb. The sequence was assembled using Canu version 2.2 (5), resulting in a single contig. As the assembly process confirmed that the genome was connected into a single contig, no further steps such as filtering were taken to improve read quality. The contig was manually circularized by removing overlapping sequences, and circularization was confirmed using Multiple Alignment using Fast Fourier Transform version 7 (6). Error correction was performed by iteratively running Medaka version 1.8.1 (https://github.com/nanoporetech/medaka) three times using the Nanopore reads. Finally, the genome was annotated by using DDBJ Fast Annotation and Submission Tool (DFAST) version 1.6.0 (7). All software was used with default settings unless otherwise specified.

The resulting circular genome of the *A. sichuanensis* had a total length of 12,144,028 bp, with a G + C content of 70.1%. It contained 11,223 coding sequences, 15 rRNA, and 103 tRNA with 4 CRISPR loci. The completeness of the genome was 99.24% by CheckM completeness check at Micromonosporaceae family via DFAST Quality Control tool (7). To investigate the potential for acarbose production, we focused on the acarbose synthesis gene, acarbose 7(IV)-phosphotransferase (*acbK*) (8). Utilizing database (UniProt) and NCBI BLAST (version 2.5.0) (9), a high-sequence similarity protein with significant similarity (*E* value, <1e^50^) to *acbK* was identified, suggesting a potential capability for acarbose production in this bacterium.

## Data Availability

The derived genomic sequence was deposited in DDBJ under accession number AP028461, and the raw reads were deposited in the Sequence Read Archive (SRA) under BioProject accession number PRJNA993787 as SRR25244093 run.
